# Organizational Health Literacy in a Hospital—Insights on the Patients’ Perspective

**DOI:** 10.3390/ijerph182312646

**Published:** 2021-11-30

**Authors:** Johanna Sophie Lubasch, Mona Voigt-Barbarowicz, Nicole Ernstmann, Christoph Kowalski, Anna Levke Brütt, Lena Ansmann

**Affiliations:** 1 Department of Health Services Research, University of Oldenburg, 26129 Oldenburg, Germany; mona.voigt-barbarowicz@uol.de (M.V.-B.); anna.levke.bruett@uol.de (A.L.B.); lena.ansmann@uol.de (L.A.); 2 Center for Health Communication and Health Services Research (CHSR), Department of Psychosomatic Medicine and Psychotherapy, University Hospital Bonn, 53127 Bonn, Germany; nicole.ernstmann@ukbonn.de; 3 German Cancer Society (DKG), 14057 Berlin, Germany; kowalski@krebsgesellschaft.de

**Keywords:** health literacy-sensitive communication, patient–professional relationship, HL-COM, information needs, patient survey

## Abstract

Health literacy-sensitive communication has been found to be an important dimension of organizational health literacy measured from the patients’ perspective. Little is known about the role of health literacy-sensitive communication in complex care structures. Therefore, our aim was to assess which hospital characteristics (in terms of process organization) and patient characteristics (e.g., age, chronic illness, etc.) contribute to better perceptions of health literacy-sensitive communication, as well as whether better health literacy-sensitive communication is associated with better patient reported experiences. Data were derived from a patient survey conducted in 2020 in four clinical departments of a university hospital in Germany. Health literacy-sensitive communication was measured with the HL-COM scale. Data from 209 patients (response rate 24.2%) were analyzed with a structural equation model (SEM). Results revealed that no patient characteristics were associated with HL-COM scores. Better process organization as perceived by patients was associated with significantly better HL-COM scores, and, in turn, better HL-COM scores were associated with more patient-reported social support provided by physicians and nurses as well as fewer unmet information needs. Investing into good process organization might improve health literacy-sensitive communication, which in turn has the potential to foster the patient–provider relationship as well as to reduce unmet information needs of patients.

## 1. Introduction

The multidimensional concept of health literacy was originally developed in the 1970s [1]. It has gained increased attention ever since the U.S. Department of Education released a report in 1993 showing that a high percentage of the country’s adult population may have insufficient literacy skills to understand written information needed to engage in health-related activity [2]. Congruent with this finding, the European Health Literacy Survey (HLS-EU) involving eight EU member states revealed that a high percentage of the population did not have adequate health literacy [3]. It defined individual health literacy as “[…] people’s knowledge, motivation and competences to access, understand, appraise, and apply health information in order to make judgments and take decisions in everyday life concerning healthcare, disease prevention and health promotion to maintain or improve quality of life during the life course” [4]. In Germany, several initiatives aiming to strengthen health literacy in the population stress the importance of individual skills and abilities in searching, understanding, evaluating, and applying health-relevant information [5,6].

In recent decades, an increase of studies concerning individual health literacy can be observed; these studies have investigated the associations between health literacy and health outcomes [7], health literacy of patients with different diseases [8,9,10], health literacy of different patient groups [11,12], health literacy interventions [13,14] and health literacy assessment tools [15]. Studies revealed that low health literacy was associated with higher hospitalization rates, greater use of emergency care, lower preventive health care use (e.g., cancer screening or vaccination) as well as an unhealthy lifestyle (e.g., physical activity), and poorer health behavior (e.g., medication adherence and self-management skills) [7,16,17,18,19,20]. Low socioeconomic status (SES), migration background, and older age were found to be associated with lower health literacy levels [21,22,23,24,25,26]. Moreover, considering that chronic conditions require a high degree of self-management [27,28] and that the ability for self-management may be impaired when health literacy is low [16,17,20], improving health literacy clearly has the potential to prevent the development of chronic diseases, or at least the occurrence of comorbidities [17,29], and to reduce the associated burdens. In line with this idea, previous results show that fostering health literacy could contribute to lower healthcare costs [30].

### 1.1. Concept of Organizational Health Literacy

Beyond the individual-based definition—of finding, understanding, evaluating, and applying health information [4]—health literacy is now understood to be a much more complex construct. Attention has shifted to the specific context in which health care is delivered, since health literacy involves the interaction with health services and other societal institutions [22]. Thus, patients’ ability to understand medical information and navigate the care process is associated with the demands placed on them by the health care system [31,32,33]. In this process, the specific organizational context in which care is delivered can help to compensate for patients’ limited health literacy [31]. Health literacy is therefore currently considered to be the product of the interaction between individuals’ capabilities and the health literacy demands and complexities of the health care system [34]. To characterize and assess organizational conditions and efforts to help patients navigate the system, the concept of health literate health care organizations—also known as organizational health literacy—has emerged [35]. Brach et al. [35] defined the following ten attributes of health literate healthcare organizations:

1. Has leadership that makes health literacy integral to its mission, structure, and operations.
2. Integrates health literacy into planning, evaluation measures, patient safety, and quality improvement.
3. Prepares the workforce to be health literate and monitors progress.
4. Includes populations served in the design, implementation, and evaluation of health information and services.
5. Meets the needs of populations with a range of health literacy skills while avoiding stigmatization.
6. Uses health literacy strategies in interpersonal communications and confirms understanding at all points of contact.
7. Provides easy access to health information and services and navigation assistance.
8. Designs and distributes print, audiovisual, and social media content that is easy to understand and act on.
9. Addresses health literacy in high-risk situations, including care transitions and communications about medicines.
10. Communicates clearly what health plans cover and what individuals will have to pay for services.

### 1.2. Organizational Health Literacy in Hospitals

The results of previous publications indicate that the ten attributes defined by Brach et al. [35] are implemented by hospitals with varying degrees of success [36,37,38]. Moreover, previous results show that organizational health literacy scores vary by hospital ownership [37,38]. However, the results remain inconclusive as to whether scores are highest in private [37,39] or university hospitals [38]. The results of the validation study of the health literate health care organization ten item questionnaire (HLHO-10) from Kowalski et al. [40] revealed that organizational health literacy is associated with the patients’ perception of having received adequate information during their hospital stay. In other studies, associations were found between HLHO-10 scores and patient satisfaction [39] as well as the healthcare professionals’ (HCP) perception of the quality of care [37]. All things considered, research on basic correlations with organizational health literacy in hospitals is limited, whereas many studies focus on interventions to foster organizational health literacy in hospitals.

### 1.3. Interventions, Barriers, and Facilitators of Organizational Health Literacy in Hospitals

Studies on interventions for fostering organizational health literacy predominantly focus on interventions supporting patients, e.g., through materials (e.g., informative flyers or brochures) or through digital support (e.g., apps) improving patient education or access to health information [41]. Other studies evaluated the effect of interventions targeting hospital staff, such as communication training, and further studies examined the effect of interventions supporting hospital governance (e.g., development and use of organizational health literacy tools) [41]. The successful implementation of organizational health literacy was found to be associated with organizational and institutional culture and leadership (e.g., priority of and commitment to health literacy), the design of the intervention (e.g., having change champions or procedures, policies, and protocols supporting health-literate practice), and available resources (e.g., time and money) [42,43,44]. Moreover, organizational health literacy was found to be fostered by high staff awareness, by knowledge and skills concerning organizational health literacy, and by the sharing of responsibilities for measures concerning organizational health literacy and practices across multiple people in the organization (e.g., using frameworks or guides) [42,45]. What has also proven to be beneficial is to tailor the intervention specifically to the needs of the organization, and to use appropriate tools for baseline assessments of current practice to inform gaps in organizational health literacy as well as for monitoring processes during implementation [45,46,47].

Tools assessing organizational health literacy are predominantly designed to be assessed by HCPs or key informants of hospitals [40,48,49]. To also allow taking the patient perspective into account, Ernstmann et al. [50] developed a scale for measuring aspects of organizational health literacy from the patients’ perspective, namely the HL-COM scale. The development phase of the scale entailed theoretical work, during which an item pool based on the ten attributes of organizational health literacy by Brach et al. [35] was generated. However, the subsequent item prioritization by cancer patients and psychometric testing resulted in a reduced item pool measuring health literacy-sensitive communication (HL-COM) as a subdimension of organizational health literacy that can be assessed by and seems to be relevant for patients [50]. Through the items, patients assess factors, such as whether they were asked if they understood information or documents, whether they were encouraged to ask questions, or whether it was ensured that they understood consent forms they signed. The HL-COM thereby measures an important aspect of organizational health literacy from the patients’ perspective. In the validation study, the instrument was found to be associated with patient enablement [50].

### 1.4. Research Question

Organizational health literacy can help to compensate for patients’ limited individual health literacy. For patients, health literacy-sensitive communication was found to be the most salient dimension of organizational health literacy that can be assessed by them [50]. Therefore, fostering health literacy-sensitive communication could potentially help to improve organizational as well as individual health literacy. However, to our knowledge, little is known about the factors influencing health literacy-sensitive communication in hospitals or about the effect of good health literacy-sensitive communication on other outputs of healthcare in hospitals. Therefore, the aim of our study was to assess which factors might contribute to better perceptions of health literacy-sensitive communication, as well as whether better health literacy-sensitive communication is associated with better patient reported experiences.

**Figure 1:**
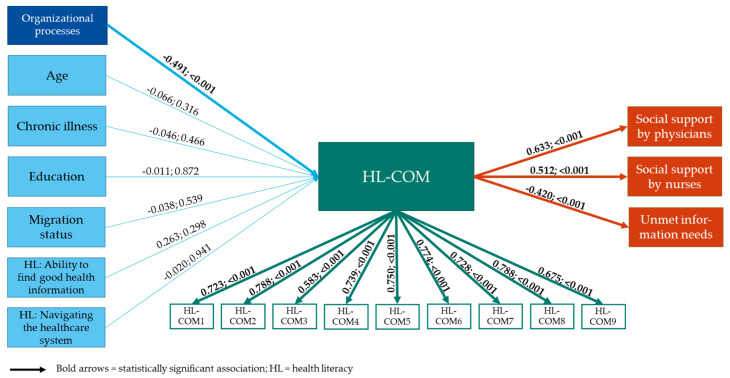
Results of the structural equation model with standardized model estimates and *p*-values.

The factors that our analysis model (see Figure 1) assumed to have an impact on health literacy-sensitive communication were selected based on the communication framework of Feldman-Stewart et al. [51]. This framework emphasizes that patient–professional communication is influenced by individual characteristics of the interacting persons as well as by the environment in which it takes place. The individual characteristics were chosen according to the characteristics that have been found to be associated to individual health literacy, namely education, migration background, age, and number of chronic diseases [17,21,22,23,24,25,26,29] (see Figure 1). Moreover, we assumed that individual health literacy itself might have an impact on health literacy-sensitive communication. As an environmental factor, process organization (e.g., coordination between wards as well as professions or waiting times) was assessed since it has already been found to be associated with patient–professional interaction in hospitals [52,53]. Previous studies assumed that professionals working in hospitals with worse process organization have fewer resources available for adequate interaction with their patients [53]. Our research questions were the following:

1. Are individual patient characteristics, in terms of education, migration background, age, number of chronic diseases, and individual health literacy associated with the patients’ perception of health literacy-sensitive communication?
2. Is the hospital’s process organization as perceived by patients associated with health literacy-sensitive communication?

The factors that our analysis model (see Figure 1) assumed to be influenced by health literacy-sensitive communication were selected based on the results of previous publications. On the one hand, the literature revealed that good patient–professional communication was key for the patient–professional-relationship [54], and that providing the patient with information fosters a supportive patient–professional relationship [55]. In our model, we therefore assumed that the provision of social support—as part of the patient–professional relationship—might be associated with health literacy-sensitive communication. On the other hand, previous publications revealed that organizational health literacy was associated with the adequacy of information patients received during their hospital stay [40]. We therefore assumed that health literacy-sensitive communication might improve the provision of health information. This resulted in the following research questions:

3. Is health literacy-sensitive communication associated with social support provided by physicians and nurses?
4. Is health literacy-sensitive communication associated with unmet information needs of patients?

## 2. Materials and Methods

Data were collected within the PIKoG study ‘As made for us—Improving professional health literacy in hospitals’ [56]. The study aims at co-designing, implementing, and evaluating a communication concept for clinical departments of a hospital. The communication concept was developed to improve health literacy at the levels of the healthcare organization, healthcare professionals, and patients. For our analysis, we used data from a patient survey conducted in 2020 prior to the implementation of the communication concept.

### 2.1. Study Site

The study was conducted in acute inpatient care at a university hospital. This non-profit general hospital in north-western Germany offers approximately 400 beds. Four out of eleven clinical departments of this hospital (oncology, gynecology, orthopedics, and visceral surgery) participated in the study. 

### 2.2. Sample

Patients were eligible for inclusion in the study if they were: (1) older than 18 years of age, (2) hospitalized for at least two nights in one of the four participating clinical departments, and (3) able to fill in the questionnaires in one of the available languages (i.e., German, English, Russian, Turkish, or Polish), either alone or with the support of a friend or relative. Moreover, the study team offered help with filling in the questionnaire to facilitate the participation of illiterate or semi-literate patients. Of 2049 eligible patients, 897 patients were asked to participate in the defined period, 473 consented to participate in the study, and 217 returned T0 and T1 questionnaires (response rate: 24.2%). Thereof, 209 completed all items relevant for the present analysis.

### 2.3. Recruitment

On their day of admission, patients treated as inpatients in the clinical departments in September through December 2020 were asked to participate in the study. Patients who had given verbal consent were provided with written study information, a consent form, and the questionnaire. Participants were asked to return the completed consent form with the address sheet and the questionnaire in sealable envelopes to mailboxes in the hospital. All patients were surveyed twice: at hospital admission (T0) and at hospital discharge (T1). Sociodemographic data as well as individual health literacy scores were assessed at T0. Data on health literacy-sensitive communication were assessed at T1. The T1 questionnaire was sent to the participants’ home address after their discharge or—if possible—handed to them in the hospital on the day of discharge. Participants were reminded to return the questionnaire twice, according to Dillman’s method [57]. 

### 2.4. Measures

Patient data were collected with questionnaires. Quality assurance during study execution was safeguarded by the standards of questionnaire development [58,59], pretesting [60], and data processing with the Teleform® software (Version 16.5.1, Electric Paper Informationssysteme GmbH, Lueneburg, Germany).

Health literacy-sensitive communication was assessed using the validated questionnaire HL-COM [50]. The HL-COM consists of 9 items rated on a four-point Likert scale ranging from 1 (‘I disagree’) to 4 (‘I fully agree’) (Cronbachs’ alpha = 0.911) [50] (for all items, see Table 1). The scale was calculated if at least 70% of items (6 items) were answered by summing up all item scores and dividing them by the number of answered items. Higher values on the HL-COM scale indicate better health literacy- sensitive communication.

The following sociodemographic and disease-related patient characteristics were collected: sex, age group, education, employment situation, type of health insurance, migration background, chronic diseases, diagnosis, and length of hospital stay. The clinical department in which the patient was treated was derived from the patient’s medical record.

The general health literacy of patients was measured using the German version of the Health Literacy Questionnaire (HLQ) [61,62]. It consists of 44 items (sample item ‘I make plans for what I need to do to be healthy‘) in 9 subscales: feeling understood and supported by healthcare providers (4 items, Cronbachs’ alpha = 0.805); having sufficient information to manage my health (4 items, Cronbachs’ alpha = 0.781); actively managing my health (5 items, Cronbachs’ alpha = 0.829); social support for health (5 items, Cronbachs’ alpha = 0.713); appraisal of health information (5 items, Cronbachs’ alpha = 0.796); ability to actively engage with healthcare providers (5 items, Cronbachs’ alpha = 0.871); navigating the healthcare system (6 items, Cronbachs’ alpha = 0.833); ability to find good health information (5 items, Cronbachs’ alpha = 0.823); and understand health information well enough to know what to do (5 items, Cronbachs’ alpha = 0.711) [61]. Subscales 1 through 5 are rated on a four-point Likert scale ranging from 1 (‘Strongly disagree’) to 4 (‘Strongly agree’), and subscales 6 through 9 are rated on a five-point Likert scale ranging from 1 (‘’Cannot do or Always difficult’) to 5 (‘Always easy’) [61], with higher values indicating higher levels of individual health literacy. The scales were calculated according to the HLQ handbook. 

Process organization during the hospital stay was measured with six items (Cronbachs’ alpha = 0.842), which were developed within the Cologne Patient Questionnaire [63,64] (sample item: ‘Here at the hospital, the right hand sometimes didn’t know what the left hand was doing.‘). The items had to be rated using four response options, ranging from ‘strongly disagree’ to ‘strongly agree’, with higher values indicating more problems with process organization. The scale was calculated if at least 70% of items (4 items) were answered by summing up all item scores and dividing them by the number of answered items.

The scales measuring the patients’ perceptions of social support from physicians as well as nurses were also developed within the Cologne Patient Questionnaire [63,64]. Both scales have already been validated (physicians: Cronbachs’ alpha = 0.924) [65] (nurses: Cronbachs’ alpha = 0.928), and each consists of three items (sample items: ‘The physicians supported me in a way that made it easier for me to deal with my illness.‘; ‘I could rely on the nurses when I had problems with my illness.‘). The items had to be rated using four response options, ranging from ‘strongly disagree’ to ‘strongly agree’, with higher values indicating more support. Each scale was calculated if at least 70% of items (2 items) were answered by summing up all item scores and dividing them by the number of answered items.

To assess the information needs of patients, they answered ‘yes’ or ‘no’ to the question of whether they would have wished to have more information concerning the following aspects: (1) ‘healthy lifestyle (diet, alcohol, smoking etc.)’; (2) ‘physical burden in everyday life’; (3) ‘mental burden in everyday life’; (4) ‘self-help groups’; (5) ‘books and brochures about their disease’; (6) ‘health promoting measures’; and (7) ‘help and care at home’. The number of times a patient stated ‘yes’ was then summed and served as a measure of unmet information needs (with higher values indicating more unmet needs).

### 2.5. Data Analysis

The associations between health literacy-sensitive communication and patient characteristics, process organization, and patient–provider relationships were analyzed within a comprehensive structural equation model (SEM) (see Figure 1). According to the HLQ handbook, missing data for the HLQ items were imputed using the expectation maximization (EM) algorithm. No further imputations were conducted since missing data for each variable were below 5%. Of the 217 patients who completed T0 and T1, 209 patients had no missing data on the variables of interest, which formed the basis for the present analysis. According to Kline et al. [66], an ideal sample size-to-parameters ratio would be 20:1. Consequently, 200 patients are sufficient to estimate a model with 10 parameters. Therefore, only two of the nine subscales of the HLQ were included, namely ‘Navigating the healthcare system’ and ‘Ability to find good health information’. The subscales were selected by conducting a prior SEM containing only HL-COM and the nine subscales of the HLQ (results not displayed). Only the HLQ subscales which showed significant associations in this prior SEM were included in the final SEM. To develop and test the SEM, the maximum likelihood estimation procedure [66] of Mplus Version 8.2 (Muthen & Muthen, Los Angeles, CA, USA) was used. The recommended thresholds were used to determine a good model fit of the SEM: root mean square error of approximation (RMSEA) 0.08–0.5, standardized root mean square residual (SRMR) < 0.5, and incremental fit indexes (comparative fit index (CFI) and Tucker–Lewis index (TLI) close to 0.90 and 0.95) [67]. IBM^®^ SPSS^®^ 26.0 (IBM Corporation, Armonk, NY, USA) was used for descriptive analysis. A significance level of α = 0.05 was chosen.

### 2.6. Ethical Considerations and Trial Registration

The study was conducted in accordance with the Declaration of Helsinki in its current version (World Medical Association (WMA), 2013). A study protocol was approved by the Ethics Committee of the Medical Faculty of Oldenburg (number: 2019-148) before the study started. All study participants were asked to provide written informed consent based on current data protection regulations. All study participants were informed that participation in the study is voluntary. All personal identifiers were pseudonymized. Data security has been approved by all institutions involved in data collection. The identifying data are stored separately from the research data. 

The study has been registered in the German Clinical Trials Register (DRKS) (trial registration number: DRKS00019830).

## 3. Results

The majority of participants in the sample were female (63.2%) and older than 50 years (62.7%) (see Table 2). Three quarters of participants reported having at least one chronic condition (76.1%), whereof 30.1% and 26.3% indicated having one or two chronic conditions, respectively (see Table 3). The most common chronic conditions were high blood pressure (30.1%), overweight/obesity (23.0%), and cancer (21.5%). Most patients were treated in the departments of orthopedics (38.8%) and gynecology (34.4%). Mean scores of the two HLQ subccales were 3.63 for ‘navigating the healthcare system’ and 3.67 for ‘ability to find good health information’ (range 1–5) (see Table 4). 

The mean scale score of HL-COM was 2.98 (range 1–4) (see Table 4), with scores for item 5 (‘People spoke slowly and clearly to me’) being the highest (see Table 1). Mean scale scores of process organization, social support by physicians, and social support by nurses were 1.76, 3.16, and 3.51, respectively (range 1–4) (see Table 4). On average, patients reported unmet needs concerning one or two of the seven aspects. 

The model fit indices indicated good model fit (see Table 5). The model results revealed no statistically significant associations between patient characteristics and HL-COM scores. All other constructs of the model showed significant associations with HL-COM scores. Worse process organization was associated with lower HL-COM scores (−0.491, *p*-value < 0.001) (see Figure 1). Moreover, higher HL-COM scores were associated with higher perceived levels of social support provided by physicians (0.633, *p*-value < 0.001) and nurses (0.512, *p*-value < 0.001). Furthermore, higher HL-COM scores were associated with fewer unmet information needs of patients (0.420, *p*-value < 0.001).

## 4. Discussion

Health literacy-sensitive communication measures are an important aspect of organizational health literacy that is relevant for patients. To our knowledge, this is the first study to investigate the role of health literacy-sensitive communication in a hospital setting from the patient view. The results of the SEM revealed that better processes organization was associated with significantly better health literacy-sensitive communication. Moreover, patients who gave higher ratings for health literacy-sensitive communication felt more supported by physicians and nurses and had fewer unmet information needs.

### 4.1. Interpretation within the Context of the Wider Literature

Concerning the association between organizational processes and health literacy-sensitive communication, we can confirm the assumptions of previous literature that problems with process organization negatively affects the patient–professional interaction [52,53]. Hence, our results are in line with the communication framework developed by Feldman-Stewart et al. [51], which suggests patient–professional interactions to be influenced by the context in which they take place. A possible explanation for this might be that physicians and nurses working in hospitals with worse process organization might have fewer ressources to interact with their patients because they are preoccupied with managing these processes [53,68]. Hence, deficits in process organization might reflect as stress and high workload among physicians and nurses [53]. 

Moreover, our results revealed that better health literacy-sensitive communication is associated with more perceived social support provided by physicians and nurses. Hence, our results confirm previous research findings that identified patient–provider communication as a key determinant for good patient–provider relationships [54], and defined the provision of information as an element contributing to a supportive patient–provider relationship [55]. Furthermore, the results of our study revealed that whenever health literacy-sensitive communication was rated better, patients’ unmet information needs were significantly lower. We thereby confirm the results from Kowalski et al. [40], who found significant associations between organizational health literacy and the adequacy of information provided by hospitals as perceived by patients. Our results imply that health literacy-sensitive communication plays an important role in patients’ information seeking process. 

Our data did not confirm our assumption that individual patient characteristics that were previously found to be associated with individual health literacy are also associated with the perception of health literacy-sensitive communication. In our sample, age, education, migration status, and chronic illness were not found to be associated with health literacy-sensitive communication. While these characteristics have been found to be associated with individual health literacy [17,21,22,23,24,25,26,29], our results are partly in line with previous findings on associations between patient characteristics and patients’ reports of communication with healthcare providers. Previous results concerning the association between age, educational level, ethnicity, native language, and comorbidities and patient–provider communication or interaction are inconsistent, and partially do not show any significant associations [52,53,68,69,70]. However, neither our data nor previous data deliver any explanation for the findings. It remains unclear how to interpret these results. Possible explanations are: (1) communication does not vary according to patient characteristics; (2) the perception of communication does not vary according to patient characteristics; (3) vulnerable patient groups are underrepresented; (4) the different patient groups in the sample did not have differing communication needs; (5) different communication needs of the patient groups were already met; or (6) a combination of these or other reasons led to the results. 

### 4.2. Strengths and Limitations 

A strength of our study is the comprehensive examination of different factors influencing health literacy-sensitive communication within one SEM. SEM has emerged as the method of choice when considering complex patterns of relationships or differences between a multitude of variables [66]. However, like any cross-sectional study, this study is not suitable for examining causality. Moreover, we conducted the study in only one hospital. Since patients were treated in four different clinical departments of the hospital, we believe that the heterogeneous group of patients participating in our study reflects the patient diversity found in hospitals. However, assessing associations between organizational health literacy and environmental factors would require analyzing several hospitals to allow comparisons between them. Furthermore, we are aware that a response rate of only one quarter is relatively low. One reason for this might be the COVID-19 pandemic, which has created uncertainty among hospital patients and reduced their willingness to participate in studies. In anticipation of this effect, we chose a recruitment period that was less impacted by the pandemic. Unfortunately, we were unable to perform a non-responder analysis. Moreover, our study is at risk of common method bias since the predictor variables and the outcome measure were both reported in the same patient survey. 

### 4.3. Implications for Practice and Research

The findings of our study suggest that better health literacy-sensitive communication contributes to fewer unmet information needs of patients. To foster health literacy-sensitive communication, communication training for healthcare professionals might be implemented, which has already been found to be a suitable measure for this purpose [41]. Special care should be taken to explain terms and abbreviations, and to combine verbal and written information (handing out verbal information in written form and verbally explaining written information), since these were the items that were rated lowest by the patients in our sample as well as in the validation sample [50]. 

Additionally, improving patient health literacy requires wider changes within healthcare organizations, as emphasized by the concept of health-literate healthcare organizations [35]. Such changes range from generating information flyers or brochures for improving patient education or access to health information, to supporting hospital governance by evaluating and managing efforts to become a health-literate healthcare organization [41]. The need for changes on the organizational level is supported by the findings of our study. The results revealed that investing in better organized processes may foster health literacy-sensitive communication. Therefore, health policy and hospital management should strive to create conditions to optimize processes in hospitals in a patient-centered way. This might be achieved by restructuring workplaces or implementing standardized work processes to foster well-organized and effective work processes as previously suggested in the context of hospital discharge [71]. 

To address the limitations of our study, future studies should be conducted in more than one hospital to allow consideration of between-hospital differences in health literacy-sensitive communication. Furthermore, the role of patient characteristics should be clarified in future studies in order to be able to address possible individual communication needs (e.g., due to chronic illness or education level) in interventions improving health literacy-sensitive communication. Moreover, efforts should be made to determine the professionals’ perspective on organizational health literacy and to compare it with the patients’ perspective to be able to assess whether patients and professionals share the same concept of health literate healthcare organizations.

## 5. Conclusions

This study provides preliminary evidence on the important role played by health literacy-sensitive communication—as a key dimension of organizational health literacy—in the healthcare of patients in hospitals. Promoting health literacy-sensitive communication may be an important measure for reducing patients’ unmet information needs. Besides communication training, improving the hospitals’ process organization might contribute to better health literacy-sensitive communication and improved relevant outputs. Furthermore, health literacy-sensitive communication is not only an important dimension of organizational health literacy but might have the potential to improve the patient–professional relationship—as demonstrated here in terms of the provision of social support. 

## Figures and Tables

**Table 1:** Items of HL-COM and frequency of response options.

			Response Options ^1^				
Item	Content		1	2	3	4	Mean Score
HL-COM1	I was made to feel that it is important for me to understand the information about my disease and treatment.	n ^a^	9	31	113	53	3.03
		%	4.3	14.8	54.1	25.4	
HL-COM2	I was asked if I understood all information or documents.	n ^a^	12	47	85	64	2.96
		%	5.7	22.5	40.7	31.6	
HL-COM3	Verbal information about my disease and treatment was additionally provided in writing.	n ^a^	25	47	67	68	2.86
		%	12.0	22.5	32.1	32.5	
HL-COM4	Terms and abbreviations were explained to me.	n ^a^	18	50	99	41	2.78
		%	8.6	23.9	47.4	19.6	
HL-COM5	People spoke slowly and clearly to me.	n ^a^	5	27	94	82	3.21
		%	2.4	12.9	45.0	39.2	
HL-COM6	I was encouraged to ask questions if I didn’t understand something.	n ^a^	10	53	78	67	2.97
		%	4.8	25.4	37.3	32.1	
HL-COM7	Written materials were additionally explained to me.	n ^a^	15	54	94	43	2.81
		%	7.2	25.8	45.0	20.6	
HL-COM8	When signing consent forms, efforts were made to ensure that I understood everything.	n ^a^	6	40	92	68	3.08
		%	2.9	19.1	44.0	32.5	
HL-COM9	My results were explained comprehensively to me.	n ^a^	6	37	96	68	3.09
		%	2.9	17.7	45.9	32.5	

^1^ 1: I disagree, 2: I somewhat disagree, 3: I somewhat agree, 4: I fully agree. ^a^ Summation of the number of respondents for each item might not equal to 209 since some patients had missing values on single items. The scale was calculated if at least 70% of items (6 items) were answered.

**Table 2:** Sociodemographic characteristics of the sample (n = 209).

		n ^a^	%
Sex	Female	132	63.2
	Male	75	35.9
	Diverse	1	0.5
	Missing	1	0.5
Age	18–29 years	18	8.6
	30–39 years	19	9.1
	40–49 years	41	19.6
	50–59 years	57	27.3
	60 years or older	74	35.4
Education	Lower secondary school education or less	41	19.6
	Intermediate secondary school education	81	38.8
	University entrance qualification	87	41.6
Migration status	Without	190	90.9
	With	19	9.1
Health insurance status	Public	164	78.5
	Public with additional private insurance	15	7.2
	Private	29	13.9
	Other	1	0.5

^a^ Due to rounding, percentages might not add up to exactly 100%.

**Table 3:** Disease and diagnosis related characteristics of the sample (n = 209).

		n ^a^	%
Number of chronic diseases	0	50	23.9
	1	63	30.1
	2	55	26.3
	3	17	8.1
	4	17	8.1
	>4	7	3.4
Chronic diseases (multiple answers possible)	High blood pressure	63	30.1
	Overweight/obesity	48	23.0
	Cancer	45	21.5
	Mental illness	32	15.3
	Cardiovascular disease	24	11.5
	Lung disease (chronic bronchitis/COPD/asthma)	18	8.6
	Arthritis or rheumatism	16	7.7
	Diabetes	13	6.2
	Kidney disease	7	3.3
	Stroke	4	1.9
	Other diseases	61	29.2
	No chronic disease	52	23.9
Clinical division in which the patient was treated	Oncology	2	1.0
	Visceral surgery	54	25.8
	Gynecology	72	34.4
	Orthopedics	81	38.8
Number of nights spent in hospital	≤3	71	33.9
	4–6	75	35.9
	7–9	39	18.7
	>9	20	8.8
	Missing	4	1.9

^a^ Due to rounding, percentages might not add up to exactly 100%.

**Table 4:** Descriptive statistics of the latent constructs and unmet information needs.

	Possible Range	Mean	SD ^1^	Observed Range	Min	Max	Cronbachs‘ α
Health literacy-sensitive communication	1–4	2.98	0.65	3.00	1.00	4.00	0.911
Process organization	1–4	1.76	0.65	3.00	1.00	4.00	0.842
Social support provided by physicians	1–4	3.16	0.70	3.00	1.00	4.00	0.924
Social support provided by nurses	1–4	3.51	0.64	2.67	1.33	4.00	0.928
Unmet information needs	0–7	1.59	1.92	7.00	0.00	7.00	-
Health literacy: Navigating the healthcare system	1–5	3.63	0.57	3.17	1.67	4.83	0.833
Health literacy: Ability to find good health information	1–5	3.67	0.58	3.20	1.60	4.80	0.823

^1^ SD = standard deviation.

**Table 5:** Fit indices of the structural equation model.

	X^2^	Df	Cronbachs’ α	RMSEA	SRMR	TLI	CFI
Threshold			≥0.7	≤0.08	≤0.08	≥0.90	≥0.90
SEM	832	521	0.911	0.048	0.070	0.920	0.926

X^2^: chi square; Df: degrees of freedom; RMSEA: root mean square error of approximation; SRMR: standardized root mean square residual; CFI: comparative fit index; TLI: Tucker–Lewis index.

## Data Availability

The data presented in this study are available on request from the corresponding author.

## References

[1] Simonds S.K. (1974). Health Education as Social Policy. Health Educ. Monogr..

[2] U.S. Department of Education (1993). Adult Literacy in America: A First Look at the Findings of the National Adult Literacy Survey.

[3] HLS-EU Consortium (2012). Comparative Report of Health Literacy in Eight EU Member States, The European Health Literacy Survey HLS-EU. http://cpme.dyndns.org:591/adopted/2015/Comparative_report_on_health_literacy_in_eight_EU_member_states.pdf.

[4] Sørensen K., Van den Broucke S., Fullam J., Doyle G., Pelikan J., Slonska Z., Brand H. (2012). Health literacy and public health: A systematic review and integration of definitions and models. BMC Public Health.

[5] Nationaler Aktionsplan Gesundheitskompetenz. https://www.nap-gesundheitskompetenz.de/.

[6] Ernstmann N., Bauer U., Berens E.-M., Bitzer E.M., Bollweg T.M., Danner M., Dehn-Hindenberg A., Dierks M.L., Farin E., Grobosch S. (2020). DNVF Memorandum Gesundheitskompetenz (Teil 1)—Hintergrund, Relevanz, Gegenstand und Fragestellungen in der Versorgungsforschung. Gesundheitswesen.

[7] Berkman N.D., Sheridan S.L., Donahue K.E., Halpern D.J., Crotty K. (2011). Low Health Literacy and Health Outcomes: An Updated Systematic Review. Ann. Intern. Med..

[8] Kilfoyle K.A., Vitko M., O’Conor R., Bailey S.C. (2016). Health Literacy and Women’s Reproductive Health: A Systematic Review. J. Women’s Health.

[9] Al Sayah F., Majumdar S.R., Williams B., Robertson S., Johnson J.A. (2013). Health Literacy and Health Outcomes in Diabetes: A Systematic Review. J. Gen. Intern. Med..

[10] Cajita M.I., Cajita T.R., Han H.-R. (2016). Health Literacy and Heart Failure: A Systematic Review. J. Cardiovasc. Nurs..

[11] Fleary S.A., Joseph P., Pappagianopoulos J.E. (2018). Adolescent health literacy and health behaviors: A systematic review. J. Adolesc..

[12] Bröder J., Okan O., Bauer U., Bruland D., Schlupp S., Bollweg T.M., Saboga-Nunes L., Bond E., Sørensen K., Bitzer E.-M. (2017). Health literacy in childhood and youth: A systematic review of definitions and models. BMC Public Health.

[13] Fernández-Gutiérrez M., Bas-Sarmiento P., Albar-Marín M.J., Paloma-Castro O., Romero-Sánchez J.M. (2018). Health literacy interventions for immigrant populations: A systematic review. Int. Nurs. Rev..

[14] Berkman N.D., Sheridan S.L., Donahue K.E., Halpern D.J., Viera A., Crotty K., Holland A., Brasure M., Lohr K.N., Harden E. (2011). Health literacy interventions and outcomes: An updated systematic review. Evid. Rep. Technol. Assess..

[15] Liu H., Zeng H., Shen Y., Zhang F., Sharma M., Lai W., Zhao Y., Tao G., Yuan J., Zhao Y. (2018). Assessment Tools for Health Literacy among the General Population: A Systematic Review. Int. J. Environ. Res. Public Health.

[16] Med J.K.P., Hasan S.M., Barnsley J., Berta W., Fazelzad R., Papadakos C.J., Giuliani M.E., Howell D. (2018). Health literacy and cancer self-management behaviors: A scoping review. Cancer.

[17] Buja A., Rabensteiner A., Sperotto M., Grotto G., Bertoncello C., Cocchio S., Baldovin T., Contu P., Lorini C., Baldo V. (2020). Health Literacy and Physical Activity: A Systematic Review. J. Phys. Act. Health.

[18] Oldach B.R., Katz M.L. (2014). Health literacy and cancer screening: A systematic review. Patient Educ. Couns..

[19] Geboers B., Brainard J.S., Loke Y.K., Jansen C.J.M., Salter C., Reijneveld S.A., De Winter A.F. (2015). The association of health literacy with adherence in older adults, and its role in interventions: A systematic meta-review. BMC Public Health.

[20] Mackey L.M., Doody C., Werner E.L., Fullen B. (2016). Self-Management Skills in Chronic Disease Management: What Role Does Health Literacy Have?. Med. Decis. Mak..

[21] Van Der Heide I., Rademakers J., Schipper M., Droomers M., Sørensen K., Uiters E. (2013). Health literacy of Dutch adults: A cross sectional survey. BMC Public Health.

[22] Sørensen K., Pelikan J.M., Röthlin F., Ganahl K., Slonska Z., Doyle G., Fullam J., Kondilis B., Agrafiotis D., Uiters E. (2015). Health literacy in Europe: Comparative results of the European health literacy survey (HLS-EU). Eur. J. Public Health.

[23] Schaeffer D., Berens E.-M., Vogt D. (2017). Health Literacy in the German Population. Dtsch. Aerzteblatt Int..

[24] Naus T. (2016). Health literacy in EU immigrants: A systematic review and integration of interventions for a comprehensive health literacy strategy. Eur. J. Public Health.

[25] Berens E.-M., Vogt D., Messer M., Hurrelmann K., Schaeffer D. (2016). Health literacy among different age groups in Germany: Results of a cross-sectional survey. BMC Public Health.

[26] Kobayashi L.C., Wardle J., Wolf M.S., Von Wagner C. (2016). Aging and Functional Health Literacy: A Systematic Review and Meta-Analysis. J. Gerontol. Ser. B.

[27] Allegrante J.P., Wells M.T., Peterson J.C. (2019). Interventions to Support Behavioral Self-Management of Chronic Diseases. Annu. Rev. Public Health.

[28] Grady P.A., Gough L.L. (2014). Self-Management: A Comprehensive Approach to Management of Chronic Conditions. Am. J. Public Health.

[29] Liu L., Qian X., Chen Z., He T. (2020). Health literacy and its effect on chronic disease prevention: Evidence from China’s data. BMC Public Health.

[30] Palumbo R. (2017). Examining the impacts of health literacy on healthcare costs. An evidence synthesis. Health Serv. Manag. Res..

[31] Rudd R.E., Renzulli D., Pereira A., Daltory L., Schwartzberg J.G., VanGeest J., Wang C. (2005). Literacy demands in health care settings: The patient perspective. Understanding Health Literacy: Implications for Medicine and Public Health.

[32] Nutbeam D. (2008). The evolving concept of health literacy. Soc. Sci. Med..

[33] Baker D.W. (2006). The meaning and the measure of health literacy. J. Gen. Intern. Med..

[34] Institute of Medicine (2013). Organizational Change to Improve Health Literacy: Workshop Summary.

[35] Brach C., Keller D., Hernandez L., Baur C., Parker R., Dreyer B., Schyve P., Lemerise A.J., Schillinger D. (2012). Ten Attributes of Health Literate Health Care Organizations.

[36] Howe C.J., Adame T., Lewis B., Wagner T. (2020). Original Research: Assessing Organizational Focus on Health Literacy in North Texas Hospitals. AJN, Am. J. Nurs..

[37] Bonaccorsi G., Romiti A., Ierardi F., Innocenti M., Del Riccio M., Frandi S., Bachini L., Zanobini P., Gemmi F., Lorini C. (2020). Health-Literate Healthcare Organizations and Quality of Care in Hospitals: A Cross-Sectional Study Conducted in Tuscany. Int. J. Environ. Res. Public Health.

[38] Hayran O., Ataç Ö., Orhan Ö. (2019). Assessment of Organizational Health Literacy in a Group of Public, Private and University Hospitals in Istanbul. J. Health Syst. Policies (JHESP).

[39] Hayran O., Özer O. (2018). Organizational health literacy as a determinant of patient satisfaction. Public Health.

[40] Kowalski C., Lee S.-Y.D., Schmidt A., Wesselmann S., Wirtz M.A., Pfaff H., Ernstmann N. (2015). The health literate health care organization 10 item questionnaire (HLHO-10): Development and validation. BMC Health Serv. Res..

[41] Zanobini P., Lorini C., Baldasseroni A., Dellisanti C., Bonaccorsi G. (2020). A Scoping Review on How to Make Hospitals Health Literate Healthcare Organizations. Int. J. Environ. Res. Public Health.

[42] Farmanova E., Bonneville L., Bouchard L. (2018). Organizational Health Literacy: Review of Theories, Frameworks, Guides, and Implementation Issues. Inq. J. Health Care Organ. Provis. Financ..

[43] Kaper M., Sixsmith J., Meijering L., Vervoordeldonk J., Doyle P., Barry M.M., De Winter A.F., Reijneveld S.A. (2019). Implementation and Long-Term Outcomes of Organisational Health Literacy Interventions in Ireland and The Netherlands: A Longitudinal Mixed-Methods Study. Int. J. Environ. Res. Public Health.

[44] Palumbo R. (2021). Leveraging Organizational Health Literacy to Enhance Health Promotion and Risk Prevention: A Narrative and Interpretive Literature Review. Yale J. Biol. Med..

[45] Meggetto E., Kent F., Ward B., Keleher H. (2020). Factors influencing implementation of organizational health literacy: A realist review. J. Health Organ. Manag..

[46] Weaver N.L., Wray R.J., Zellin S., Gautam K., Jupka K. (2012). Advancing Organizational Health Literacy in Health Care Organizations Serving High-Needs Populations: A Case Study. J. Health Commun..

[47] Jessup R.L., Osborne R.H., Buchbinder R., Beauchamp A. (2018). Using co-design to develop interventions to address health literacy needs in a hospitalised population. BMC Health Serv. Res..

[48] Dietscher C., Lorenc J., Pelikan J.M. (2015). Piloting of the “Self-Assessment Tool to Investigate the Organizational Health Literacy of Hospitals” following the Vienna Concept of a Health Literate Health Care Organization.

[49] Trezona A., Dodson S., Osborne R.H. (2018). Development of the Organisational Health Literacy Responsiveness (Org-HLR) self-assessment tool and process. BMC Health Serv. Res..

[50] Ernstmann N., Halbach S., Kowalski C., Pfaff H., Ansmann L. (2017). Measuring attributes of health literate health care organizations from the patients’ perspective: Development and validation of a questionnaire to assess health literacy-sensitive communication (HL-COM). Z. Evidenz Fortbild. Qual. Gesundh..

[51] Feldman-Stewart D., Brundage M., Tishelman C. (2005). A conceptual framework for patient-professional communication: An application to the cancer context. Psycho-Oncology.

[52] Ansmann L., Wirtz M.A., Kowalski C., Pfaff H., Visser A., Ernstmann N. (2014). The impact of the hospital work environment on social support from physicians in breast cancer care. Patient Educ. Couns..

[53] Lubasch J., Lee S., Kowalski C., Beckmann M., Pfaff H., Ansmann L. (2021). Hospital Processes and the Nurse-Patient Interaction in Breast Cancer Care. Findings from a Cross-Sectional Study. Int. J. Environ. Res. Public Health.

[54] Honavar S.G. (2018). Patient–physician relationship—Communication is the key. Indian J. Ophthalmol..

[55] Caplan G. (1974). Support Systems and Community Mental Health, Lectures on Concept Development.

[56] Lubasch J.S., Voigt-Barbarowicz M., Lippke S., De Wilde R.L., Griesinger F., Lazovic D., Villegas P.C.O., Roeper J., Salzmann D., Seeber G.H. (2021). Improving professional health literacy in hospitals: Study protocol of a participatory codesign and implementation study. BMJ Open.

[57] Dillman D.A. (1978). Mail and Telephone Surveys: The Total Design Method.

[58] Bradburn N.M., Sudman S., Wansink B. (2004). Asking questions: The Definitive Guide to Questionnaire Design for Market Research, Political Polls, and Social and Health Questionnaires.

[59] Groves R.M., Fowler F.J., Couper M.P., Lepkowski J.M., Singer E., Tourangeau R. (2011). Survey Methodology.

[60] Prüfer P., Rexroth M., Mohler P.P. (2000). Two-phase pretesting. Querschnitt: Festschrift für Max Kaase.

[61] Osborne R.H., Batterham R.W., Elsworth G.R., Hawkins M., Buchbinder R. (2013). The grounded psychometric development and initial validation of the Health Literacy Questionnaire (HLQ). BMC Public Health.

[62] Nolte S., Osborne R.H., Dwinger S., Elsworth G.R., Conrad M.L., Rose M., Härter M., Dirmaier J., Zill J.M. (2017). German translation, cultural adaptation, and validation of the Health Literacy Questionnaire (HLQ). PLoS ONE.

[63] Ansmann L., Kowalski C., Pfaff H. (2016). Ten Years of Patient Surveys in Accredited Breast Centers in North Rhine-Westphalia. Geburtshilfe Frauenheilkd..

[64] Pfaff H. (2003). The Cologne Patient Questionnaire (CPQ): Development and Validation of a Questionnaire to Assess Patient Involvement as a Therapist.

[65] Ommen O., Wirtz M.A., Janssen C., Neumann M., Ernstmann N., Pfaff H. (2010). Validation of a theory-based instrument measuring patient-reported psychosocial care by physicians using a multiple indicators and multiple causes model. Patient Educ. Couns..

[66] Kline R.B. (2016). Principles and Practice of Structural Equation Modeling.

[67] Schumacker R.E., Lomax R.G. (2016). A Beginner’s Guide to Structural Equation Modeling.

[68] Ansmann L., Kowalski C., Ernstmann N., Ommen O., Pfaff H. (2012). Patients’ perceived support from physicians and the role of hospital characteristics. Int. J. Qual. Health Care.

[69] Dad T., Tighiouart H., Lacson E., Meyer K.B., Weiner D.E., Richardson M.M. (2018). Hemodialysis patient characteristics associated with better experience as measured by the In-center Hemodialysis Consumer Assessment of Healthcare Providers and Systems (ICH CAHPS) survey. BMC Nephrol..

[70] Van Der Veer S.N., Arah O.A., Visserman E., Bart H.A.J., De Keizer N.F., Abu-Hanna A., Heuveling L.M., Stronks K., Jager K.J. (2012). Exploring the relationships between patient characteristics and their dialysis care experience. Nephrol. Dial. Transplant..

[71] Nowak M., Swora M., Karbach U., Pfaff H., Ansmann L. (2021). Associations between hospital structures, processes and patient experiences of preparation for discharge in breast cancer centers: A multilevel analysis. Health Care Manag. Rev..

